# Early esophageal cancer diagnosis in Central Oromia, Ethiopia: patient and provider perspectives on barriers and facilitators

**DOI:** 10.1038/s41598-026-66791-9

**Published:** 2026-08-25

**Authors:** Teresa Kisi Beyen, Edom Seife, Biniyam Tefera Deressa, Girma Taye, Adamu Addissie

**Affiliations:** 1 Department of Public Health, College of Health Science, Arsi University, Asella, Ph.D. student @SPH, AAU, Ethiopia, Ethiopia; 2 Global & Planetary Health Working Group, Institute of Medical Epidemiology, Biostatistics and Informatics, Martin Luther University Halle-Wittenberg, Halle (Saale), Germany; 3 Clinical Oncologist, College of Health Science, Addis Ababa University, Addis Ababa, Ethiopia; 4 Clinical Oncology, Adama Hospital Medical College, Adama, Ethiopia; 5 Department of Preventive Medicine, School of Public Health, College of Health Science, Addis Ababa University, Addis Ababa, Ethiopia

**Keywords:** Esophageal cancer, Early Diagnosis, Diagnostic delay, Qualitative research, Ethiopia, Healthcare access, Cancer, Health care, Medical research, Oncology

## Abstract

In Ethiopia, 70–90% of esophageal cancer patients are diagnosed at advanced stages (III and IV), with the highest burden in Oromia’s Arsi and Bale zones. Despite the burden of esophageal cancer, little is known about the factors influencing its early diagnosis in Central Oromia. This qualitative study sought to address this gap by exploring barriers and facilitators from the perspectives of both patients diagnosed at late stages and healthcare providers. Between August 17 and September 26, 2025, in-depth interviews were conducted using an exploratory case study design with 13 late-stage esophageal cancer patients and 11 healthcare providers across four Ethiopian hospitals. Participants were recruited from the hospitals using criterion sampling, and all were residents of Central Oromia, specifically Arsi, West Arsi, Bale, East Bale, and East Shewa, until data saturation was achieved. Inductive thematic analysis was performed using MAXQDA (version 24.4.1). Five main themes emerged: patient-related factors, health system-related factors, policy and strategy-related factors, emotional and ethical challenges in care, and research and evidence gaps. Late presentation and delayed health-seeking, reliance on traditional and religious care, Cancer-related fear, stigma, and fatalistic beliefs, limited awareness and education, financial constraints, geographic inaccessibility, misdiagnosis, referral system dalliance and complexities, diagnostic service limitations, workforce shortages, insufficient government support, absence of contextual screening frameworks, and research and evidence gaps were barriers to early esophageal cancer. In contrast, facilitators comprised family influence and support, health insurance coverage, and multidisciplinary and integrated cancer care. Delayed diagnosis in Central Oromia resulted from complex, multi-level factors. Addressing these barriers requires a comprehensive strategy including public awareness campaigns, healthcare provider training, improved referral systems, and expanded access to affordable, decentralized diagnostic services. Context-specific interventions are essential to facilitate earlier diagnosis and improve survival outcomes for esophageal cancer patients in central Oromia and similar Ethiopian settings.

## Introduction

Sustainable Development Goal 3 (SDG 3), target 3.4, underscores the commitment to ensuring healthy lives and promoting well-being for all ages, with a specific sub-target addressing the reduction of premature mortality from non-communicable diseases (NCDs) by one-third by 2030^1,2^. Globally in 2022, esophageal cancer ranked as the 11th leading cause of cancer-related illness and the 7th leading cause of cancer death^3^. In Ethiopia, it ranked 14th for cancer morbidity and 10th for cancer mortality^4^.

From NCD, Esophageal cancer is a significant global health concern, particularly in low- and middle-income countries where early diagnosis remains a challenge^4–7^. In Ethiopia, esophageal cancer is one of the leading causes of cancer-related morbidity^8–12^ and mortality^4,9^, often diagnosed at advanced stages due to limited awareness, healthcare access, poorly structured referrals and diagnostic facilities^9,10,12,13^.

Ethiopia is found in East Africa’s high-risk corridor for squamous cell carcinoma of the esophagus^3,14,15^. The incidence of the disease is increasing in the country while there is low attention and facilities for early screening and diagnosis, management, and research in the area^4,16–20^. Higher incidence of the tumor was reported in Oromia Regional State, particularly in the two zones of Arsi and Bale, which included the area where the study was conducted^8,10,11,21^.

The pathway to cancer treatment can be broken down into three key time intervals. First is the patient interval, which spans from the initial onset of symptoms to the patient’s first visit with a healthcare professional. Second is the diagnostic interval, covering the period from that first presentation to receiving a formal diagnosis. Third is the treatment interval, defined as the time between diagnosis and the initiation of treatment^22,23^. Early esophageal cancer diagnosis refers to detecting cancerous or precancerous changes in the esophagus before the disease has grown deep into the esophageal wall or spread to lymph nodes or other organs (i.e., at Stage I or Stage II)^24–26^.

Early detection and accurate diagnosis are critical strategies for improving the treatment outcomes of esophageal cancer^25^. Early detection involves identifying cancer in symptomatic individuals and screening asymptomatic high-risk groups. Early diagnosis and timely treatment are equally critical. One-third of cancers can be cured when detected early and managed appropriately. This plan prioritizes strengthening diagnostic and treatment services, ensuring accessibility, affordability, and quality care^27^.

As the studies pinpointed, the survival of esophageal cancer patients varied from time to time, country to country, and from region to region which was linked to the stage at which the patients were diagnosed^7,28,29^. Esophageal cancer symptoms appear late, leading to delayed diagnosis. While advanced-stage prognosis is very poor (less than 20% survival at 5 years), early diagnosis enables curative treatment with excellent outcomes (over 80% survival)^30^. A review by Xu et al. (2020) found that the 5-year survival rate for esophageal cancer patients diagnosed at early stages (stage I & II) was 90%, compared to only 6–15% for those diagnosed at advanced stages (stage III & IV)^31^.

In Ethiopia, the studies showed that about 70–90% of the patients visited health institutions with advanced stages (i.e., stages III & IV) of esophageal cancer^9,10,12,13,32^ which leads to low survival of the patients. In line with this the median time from first recognition of the disease to diagnosis was reported to be more than two months^32^.

According to the Ethiopian Ministry of Health, the provision of timely and accurate diagnoses is fundamental to effective patient care and treatment planning. Nevertheless, disparities in access to diagnostic services remain, especially among underserved populations. Bridging these gaps necessitates a coordinated approach that includes strengthening healthcare workforce capacity, improving infrastructure, and fostering innovation and collaboration across the diagnostics ecosystem^33^. This is essential because, for esophageal cancer specifically, early and timely diagnosis enables curative treatments to be more successful, which is critical for improving patient outcomes. Accordingly, enhancing early detection alongside treatment was a key objective of Ethiopia’s national cancer control plan^34^.

Despite the rising incidence of esophageal cancer in Ethiopia and the high rate of advanced-stage presentation, the factors influencing early diagnosis remain unexplored. Thus, the objective of this study was to explore barriers and facilitators to early esophageal cancer diagnosis in Central Oromia, Ethiopia, from the perspectives of patients diagnosed at late stages and healthcare providers. Understanding the factors that influence early diagnosis is essential for improving patient outcomes and reducing the burden of esophageal cancer in the region. This study sought to answer the following research question: ‘What factors influence early diagnosis of esophageal cancer in Central Oromia, Ethiopia?’ By identifying barriers and facilitators, this research aims to inform strategies for early intervention, guide policymakers and healthcare planners, and support the development of locally appropriate, context-specific interventions. Additionally, the findings may serve as a baseline for future research.

## Research question

What are the factors influencing patients’ early diagnosis of esophageal cancer?

### Methods and materials

#### Study setting

The study was conducted among patients from five zones in Central Oromia, Arsi, West Arsi, Bale, East Bale, and East Shewa, who visited four hospitals: Asella Referral and Teaching Hospital, Meda Walabu University General Hospital, Adama Hospital Medical College, and Tikur Anbassa Specialized Hospital. Healthcare providers working in these hospitals were also included in the study. Details of the zones are described elsewhere^35^.

#### Study approach and period

This study was conducted within an interpretivist paradigm, which assumes that reality is subjective and can best be understood through individuals’ experiences and the meanings they attribute to them^36–38^. An exploratory case study design was employed to gain an in-depth understanding of patients’ and healthcare providers’ experiences, perceptions, and perspective on the factors influencing the early diagnosis of esophageal cancer in Central Oromia, Ethiopia. An exploratory case study was considered appropriate because it enables a comprehensive investigation of a contemporary phenomenon within its real-life context, particularly when the boundaries between the phenomenon and its context are not clearly evident^38,39^. The research inquiry guiding this study was: why do patients delay their first visit to a health institution until esophageal cancer has reached late stages (III and IV)? By focusing on this specific case, the study aimed to uncover the multifaceted factors, ranging from individual patient experiences and healthcare provider practices to systemic and infrastructural challenges that influence the timeliness and accuracy of early esophageal cancer diagnosis. The case study design facilitated the use of multiple groups, including patients and health care providers, to capture a holistic and rich understanding of the factors influencing early esophageal cancer diagnosis. Through this comprehensive exploration, the study sought to generate insights that could inform improvements in diagnostic processes and ultimately contribute to better clinical outcomes for esophageal cancer patients. The study was conducted from August 17 to September 26, 2025, following a case study approach.

#### Source population and eligibility

The study targeted two main groups: healthcare providers working in oncology clinics, and adult patients aged ≥ 18 years with endoscopically and/or histologically confirmed advanced-stage esophageal cancer (stage III or IV). Patients were recruited from five zones in Central Oromia, Ethiopia, having initially sought diagnosis or care at one of four selected hospitals: Asella Referral and Teaching Hospital, Meda Walabu University General Hospital, Adama Hospital Medical College, and Tikur Anbassa Specialized Hospital. These facilities were chosen because they provide referral and/or therapy services for esophageal cancer patients in the study area. Patients who were critically ill and unable to participate in interviews were excluded. Healthcare providers were recruited from the same four hospitals. The providers were selected based on their involvement in esophageal cancer care, including physicians and nurses with at least one year of working experiences.

#### Participant recruitment and size

Maximum variation sampling was employed to select esophageal cancer patients and healthcare providers with diverse backgrounds and experiences, with the aim of capturing a broad range of perspectives relevant to the research objectives. This approach was used until data saturation was reached both for patients and healthcare providers, that is, the point at which no new or relevant information was emerging from additional interviews. The determination of the sample size was guided by the principle of capturing variability across key characteristics, as well as achieving saturation. Data saturation refers to the buildup of qualitative data to the idea that a sense of conclusion is achieved because additional data offer duplicate information^40^.

Accordingly, 13 patients and 11 healthcare providers participated in the study. The patients were selected to ensure variation across several important dimensions, including gender mix, stage of cancer (Stage III and IV), and place of residence (including both urban and rural settings). Similarly, for healthcare providers, we included nurses and physicians working in the oncology clinic with at least one year work experience. The selection of this mix followed the framework proposed by Mosera A. and Korstjens I., which recommends a diverse yet manageable number of participants for in-depth interviews. This composition was intended to capture the multidimensional viewpoints of clinical staff directly involved in the diagnosis of esophageal cancer^40^.

### Data collection

Face-to-face in-depth interviews were conducted with selected esophageal cancer patients and healthcare providers using open-ended guiding questions. Interviews were audio-recorded to ensure accurate capture of participants’ narratives. The interview guide, comprising approximately 20 items, was designed based on participants’ role, to elicit both patients’ experiences and healthcare providers’ perspectives. The guide included open-ended questions, probes, and prompts designed to encourage deeper responses. Organized according to the research objectives, the guide progressed from broad to specific and sensitive questions, covering topics related to early and late diagnosis and the factors influencing early esophageal cancer diagnosis. Patient interviews were conducted in a private room at the oncology unit, which had been designated for this purpose following consultation with the head of the oncology unit. Interviews with healthcare providers were conducted in their offices during their off-duty hours, to minimize disruption to clinical services.

At each hospital, one trained nurse with clinical experience in oncology, assisted by a facilitator and supervised by an oncology resident, conducted the in-depth interviews. Prior to each interview, the purpose of the study and discussion topics were clearly explained to participants. Interviews lasted approximately 40 to 60 min. After each session, data collectors engaged in self-reflection to evaluate and improve the interviewing process.

### Data processing, management and analysis

The audio-recorded interviews were transcribed verbatim and translated into English while preserving the original meaning and context of participants’ responses. The transcripts were verified against the audio recordings through repeated listening and careful reading to ensure transcription accuracy.

Data were analyzed using an inductive thematic analysis following Braun and Clarke’s six-phase approach^36,41–43^. This approach was selected because it enabled themes to develop directly from participants’ experiences and perspectives rather than being informed by pre-existing theoretical concepts^36,42–44^. Initially, the researchers familiarized themselves with the transcripts through repeated reading. The transcripts were then imported into MAXQDA 24.4.1, where an initial round of manual coding was conducted to deepen familiarity with the data and identify preliminary concepts. Subsequently, meaningful units of text were systematically coded line by line across all transcripts. The software was then used to refine the coding frame, apply it consistently across all transcripts, and facilitate theme development, data retrieval, and documentation of the analytical process. The first author conducted the initial coding, while the co-authors reviewed the coding framework and emerging themes during regular meetings. Any differences in interpretation were discussed until consensus was reached, and the final coding framework and themes were approved by all authors. Similar codes were then grouped into preliminary themes, which were reviewed and refined through an iterative process to ensure they accurately represented the dataset. The final themes were subsequently defined and named before being organized into a coherent narrative supported by representative participant quotations.

A comprehensive codebook was developed iteratively throughout the analysis and consisted of hierarchical coding structures, including parent codes, child codes, and emerging themes. The evolving codebook and thematic structure were regularly reviewed and refined during team discussions to ensure consistency and credibility throughout the analysis. This collaborative audit process enhanced methodological rigor and minimized individual researcher bias.

### Trustworthiness

The interview guide was initially developed in English to align with the study’s objectives and core areas of inquiry. It was then translated into Amharic and Afaan Oromo by bilingual experts familiar with both medical terminology and the local cultural context. To ensure the accuracy and consistency of the translations, a back-translation into English was conducted by an independent language expert. This process helped identify and correct any discrepancies, ensuring the translated guides retained their intended meaning and cultural relevance.

**Credibility** was enhanced through prolonged engagement with participants, iterative data collection and analysis, which involved allocating sufficient time for interviews and building rapport with participants to encourage open and honest responses^45,46^. Data triangulation was applied by collecting information from different participant groups (patients and healthcare providers), across varied times and settings. Investigator triangulation, involving multiple researchers in coding, analysis, and interpretation, reduced individual bias. We conducted member checking with healthcare providers by sharing interview transcripts for verification. Providers were contacted via telephone and in person, and were asked to review their transcripts for accuracy and clarity. Feedback was incorporated into the final transcripts. Member checking was not conducted with patients due to logistical constraints and the sensitive nature of their condition. Regular discussions were conducted among the research team during coding and theme development, and the use of representative quotations to support the findings.

**Transferability** was supported through thick description of the study context, participants’ backgrounds, and the healthcare environment. A detailed description of the study setting, participant characteristics, sampling procedures, and the research context were conducted to enable readers to assess the applicability of the findings to similar settings^45,46^.

**Dependability** was ensured through a well-maintained audit trail, which documented key decisions and methodological steps throughout the study^45,46^. This trail was made available during the proposal defense, ethical reviews, academic seminars, and peer evaluation for publication, enhancing transparency and accountability.

**Conformability** was strengthened through reflective documentation^45,46^. Researchers kept field notes and reflective journals detailing interview settings, participant interactions, and contextual factors. These reflections continued throughout the transcription and analysis processes, helping ensure that interpretations were grounded in the data rather than influenced by researcher bias. Together, these strategies established a rigorous and transparent research process that produced credible and meaningful insights into the factors influencing early diagnosis of esophageal cancer from the perspectives of patients and healthcare providers.

### Ethical approval and consent to participant

Ethical approval was obtained from the Institutional Review Board (IRB) of the Oromia Health Bureau (Protocol No. BFO/H2/032/17) and the IRB of the College of Health Sciences, Addis Ababa University (Protocol No. 088/24/SPH). The study was conducted in accordance with national and institutional ethical guidelines and the principles of the Declaration of Helsinki.

Verbal informed consent was obtained from all participants after they had received information about the study objectives, procedures, and their role in the research. Participation was voluntary, and participants were informed of their right to decline to answer any question or withdraw from the study at any time without any consequences.

To ensure confidentiality, no personal identifiers were collected. Instead, unique identification codes were assigned to participants and used throughout data collection and analysis. Interviews were conducted in a private setting, and audio recordings were securely stored in a password-protected folder accessible only to the research team. The recordings were permanently deleted after completion and publication of the study. Participants who were critically ill or had discontinued medical follow-up were advised and encouraged to re-engage with appropriate healthcare services.

## Results

### Socio-demographic and participants characteristics

#### Patients’ socio-demographic characteristics

Seven of the total patients interviewed were male (53.85%), and eleven of them were from rural areas (84.62%). Twelve patients were married (92.30%), and eight of them were Muslim followers (61.54%). Twelve of them had no formal education (92.30%), and eleven of them were farmers (84.64) while the other two were merchant (7.69%) and animal husbandry (7.69%). Clinically, seven of the patients interviewed were diagnosed with Stage III (53.85%) esophageal cancer. The age of the patients ranged from 40 to 84 years, with a median age of 55 years (IQR = 12) (Tables 1 and 2).

**Table 1:** Study participants characteristic (*n* = 24), 2026.

Participants code	Age	Residence	Educational status	Work status	Religion	Marital status	Sex	Stage of cancer	Type of participant
P#1	43	Urban	No formal school	Farmer	Muslim	Widowed	Female	III	Patient
P#2	53	Rural	No formal school	Merchant	Orthodox	Married	Female	IV	Patient
P#3	50	Rural	No formal school	Farmer	Muslim	Married	Female	III	Patient
P#4	84	Rural	No formal school	Farmer	Orthodox	Married	Male	IV	Patient
P#5	30	Urban	BSc. Degree	Nursing	Muslim	Single	Male	NA	Nurse
P#6	34	Urban	BSc. Degree	Nursing	Orthodox	Married	Male	NA	Nurse
P#7	29	Urban	BSc. Degree	Physician (GP)	Muslim	Married	Female	NA	Physician
P#8	65	Rural	No formal school	Farmer	Muslim	Married	Male	IV	Patient
P#9	40	Rural	No formal school	Animal husbandry	Muslim	Married	Female	III	Patient
P#10	60	Rural	Primary	Farmer	Orthodox	Married	Female	IV	Patient
P#11	56	Rural	No formal school	Farmer	Muslim	Married	Male	III	Patient
P#12	35	Urban	BSc. Degree	Nursing	Protestant	Married	Male	NA	Nurse
P#13	23	Urban	BSc. Degree	Nursing	Orthodox	Married	Male	NA	Nurse
P#14	37	Urban	Oncologist	Physician	Protestant	Married	Male	NA	Physician
P#15	82	Urban	No formal school	Farmer	Orthodox	Married	Male	III	Patient
P#16	55	Rural	No formal school	Farmer	Muslim	Married	Female	III	Patient
P#17	54	Rural	No formal school	Farmer	Muslim	Married	Male	III	Patient
P#18	30	Urban	BSc. Degree	Nursing	Muslim	Single	Male	NA	Nurse
P#19	31	Urban	Masters	Adult Nursing	Muslim	Married	Male	NA	Nurse
P#20	38	Urban	Oncologist	Physician	Orthodox	Single	Male	NA	Physician
P#21	54	Rural	No formal school	Farmer	Muslim	Married	Male	IV	Patient
P#22	83	Rural	No formal school	Farmer	Orthodox	Married	Male	IV	Patient
P#23	45	Urban	Master	Adult Nursing	Orthodox	Married	Female	NA	Nurse
P#24	30	Urban	BSc. Degree	Physician (GP)	Orthodox	Single	Male	NA	Physician

**Table 2:** Patients’ Socio-demographic characteristics (*n* = 13), 2026.

Characteristics	Number (%)					
Gender						
Male	7 (53.85)					
Female	6 (46.15)					
Residence						
Rural	11 (84.62)					
Urban	2 (15.38)					
Marital status						
Married	12 (92.30)					
Widowed	1 (7.70)					
Religion						
Muslim	8 (61.54)					
Orthodox	5 (38.46)					
Education status						
No formal School	12 (92.30)					
Primary school	1 (7.70)					
Work Status						
Farmer	11 (84.62)					
Merchant	1 (7.69)					
Animal Husbandry	1 (7.69)					
Cancer stage						
Stage III	7 (53.85)					
Stage IV	6 (46.15)					

Age distribution in year						
Minimum	1st quartile	Median	Mean	3rd quartile	Max	IQR
40.0	53.0	55.0	59.92	65.0	84.0	12.0

### Health care providers’ socio-demographic characteristics

Among the healthcare providers (HCP) interviewed, nine were male (81.82%), and seven were married (63.64%). Five HCP were Orthodox Christian while the others were Muslim (36.36%) and Protestant (18.18%). Seven of the HCP interviewed held Bachelor of Science (BSc) degree (63.64%), while two had a Master’s degree (18.18%) and two were oncologist (18.18%). Professionally, seven of the HCP participated were nurses (63.64%), whereas four were physicians (36.36%). The age of the healthcare providers ranged from 30 to 45 years, with a median age of 32.9 years (IQR = 6years) (Tables 1 and 3).

**Table 3:** Health care providers’ Socio-demographic characteristics (*n* = 11), 2026.

Characteristics	Number (%)					
Gender						
Male	9 (81.82)					
Female	2 (18.18)					
Marital status						
Married	7 (63.64)					
Single	4 (36.36)					
Religion						
Muslim	4 (36.36)					
Orthodox	5 (45.46)					
Protestant	2 (18.18)					
Education status						
BSc. Degree	7 (63.64)					
Masters	2 (18.18)					
Specialist (oncologist)	2 (18.18)					
Work Status						
Nursing	7 (63.64)					
Physician	4 (36.36)					

Age distribution in year						
Minimum	1st quartile	Median	Mean	3rd quartile	Max	IQR
40.0	30.0	32.91	59.92	36.0	45.0	6.0

### Themes identified

**Fig. 1:**
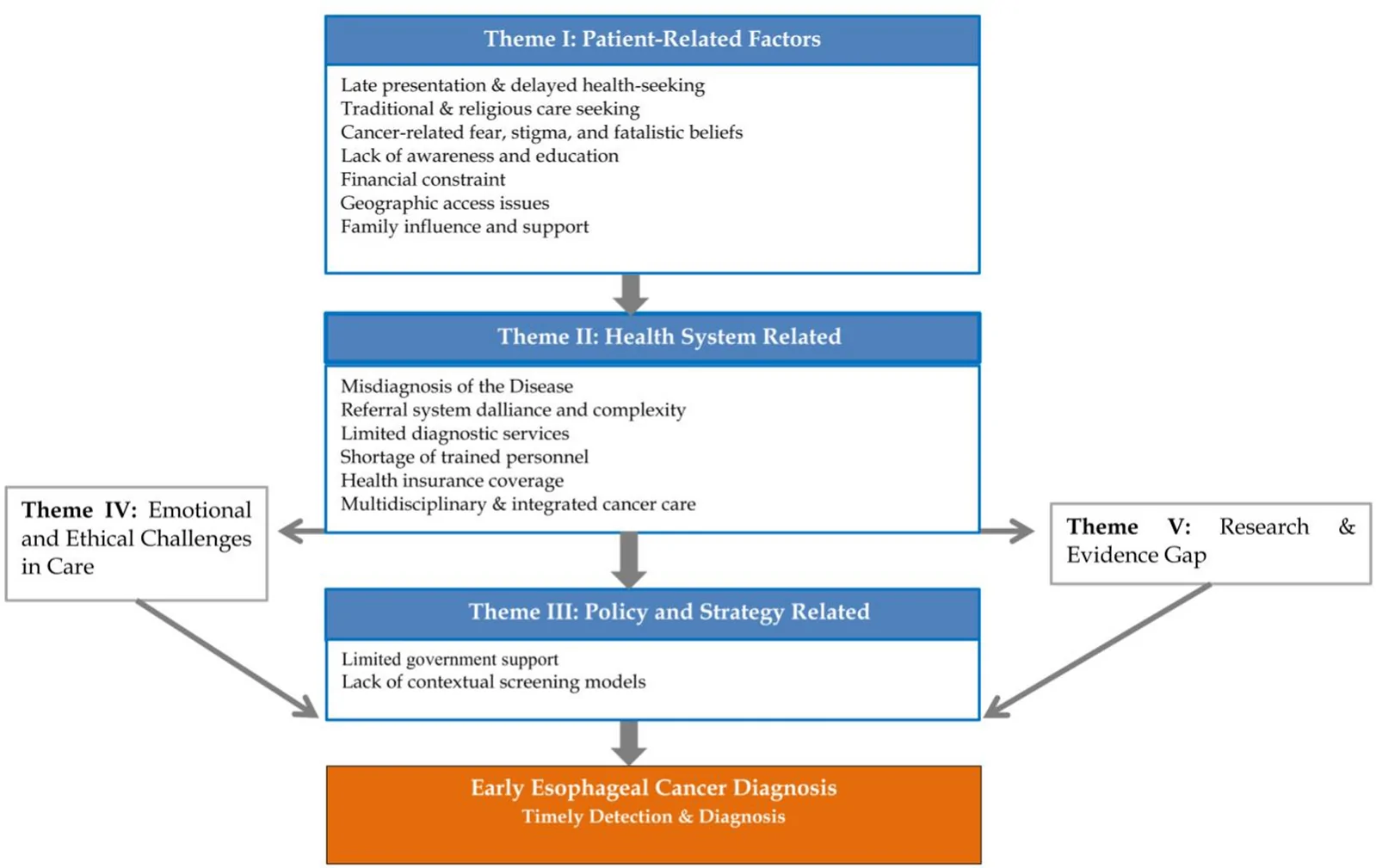
Themes identified for factors influencing early esophageal cancer diagnosis.

Thematic analysis of interview data from esophageal cancer patients and healthcare professionals identified several interconnected factors that influenced early diagnosis, serving as either barriers or facilitators. These factors were identified across patient, health system, policy and strategy, research and evidence, and emotional and ethical challenge levels. At the patient level, late presentation and delayed health-seeking behavior, reliance on traditional and religious healing practices, cancer-related fear, stigma, and fatalistic beliefs, low awareness and educational attainment, financial barriers, and limited geographic access to healthcare were identified as barriers to early esophageal cancer diagnosis. In contrast, family influence and support acted as facilitators of early diagnosis. At the health system level, early diagnosis of esophageal cancer was hindered by misdiagnosis, a referral system dalliance and complexity, limited diagnostic services, and a shortage of trained healthcare workers, while health insurance coverage and multidisciplinary and integrated cancer care served as facilitators. Additionally, gaps at the policy and strategic level, such as insufficient government support and the lack of locally appropriate screening and diagnostic models, further hindered the early diagnosis of esophageal cancer. Emotional and ethical challenges in care, as well as a lack of research and evidence, emerged as additional themes influencing the early diagnosis of esophageal cancer. Verbatim quotations that capture the essence of each sub-theme have been incorporated into the Results section to validate and ground the findings in participants’ own voices. In addition, we summarized the themes, sub-themes, and representative quotations to improve the clarity and presentations of the results (Fig. 1; Table 4).

**Table 4:** Summary of themes, sub-themes and representative quotations.

Main themes	Sub-themes	Representative quotations
Theme I: Patient-related factors	Late presentation and delayed health-seeking	“…The reason I failed to realize it quickly was just since I am merchant, I was busy…Within those three months, I didn’t know that it was this disease…” (P#2, 53years, Female, EC Stage IV patient)
	Traditional and religious care seeking	“…Some even said they tried traditional healing…They say they put some tube plant (Shanbaqqoo) through the mouth down to the esophagus and then insert hot metal inside to burn it…We’ve seen two or three such cases… It even caused a fistula…it connected the esophagus and the trachea (Tracheoesophageal fistula)… They also apply burning on their chest, called ‘Koobaa’…” [P#19, Male, 38years, Physician]
	Cancer-related fear, stigma, and fatalistic beliefs	“…When cancer is mentioned, people become frightened…There is a strong belief that anyone who has cancer will inevitably die…Some patients run away immediately when they hear the word…Almost all of them hide it… They ask us to keep it secret…” [P#5, Male, 30years, Nurse]
	Lack of awareness and education	“….Do I know anything? They are the ones who know… They told me this is the kind of disease that brings people to the grave…I didn’t know this disease before. Even now, I don’t understand it well… ” [P#8, Male, 65years, EC Stage IV patient]
	Financial constraint	“…I don’t even have money for medical tests… we sold the land, did other things… people who have seen this situation pitied me and asked for help….” [P#1, Female, 43years, EC Stage III patient]
	Geographic access issues	“…CT scan wasn’t available… so we used to refer patients to a town called **Dodola.** There’s also transport cost involved. Even there, accurate staging is not done properly…” [P#20, Male, 38years, Oncologist]
	Family influence and support	“…It was actually one of my children, who told me to go…They said, you can’t even grind grain anymore… So I followed their advice and came to stay with my children … you’re making your children cry… The children don’t spare any effort… They said it won’t be left behind…Only one of my children refused. …” [P#10, Female, 60years, EC Stage IV patient]
Theme II: Health system related	Misdiagnosis of the disease	“…I kept being told it was just gastritis… Whenever they listen to me with a stethoscope, they say it’s gastritis. Wherever they press on my body, they say it’s gastritis …” [P#1, Female, 43years, EC Stage III patient]
	Referral system dalliance and complexity	“…The hospital in Gobessa referred me to Asella. Then Asella referred me here, to Adama Hospital… then to Tikur Anbessa Hospital…To find out what the disease was… After going through all this… I finally found something…” [P#8, Male, 65years, EC Stage IV Patient]
	Limited diagnostic services	“…The availability of diagnostic centers is currently limited in the country… Diagnoses like CT scans are not found…It exists, but sometimes it doesn’t work…The diagnosis called Immunohistochemistry (IHC) … is often sent outside for identification…” [P#13, Male, 23years, Nurse]
	Shortage of trained personnel	“…Honestly, the hospital here is in poor condition … There is no doctor who can properly identify the illness…” [P#21, Male 54years, EC Stage IV patient]
	Health insurance coverage	“…If the diagnosis system is available, insurance will cover it… Most of the time, they are not available…” [P#20, Male, 38years, Oncologist]
	Multidisciplinary and integrated cancer care	“…We cannot work alone…We need pathologists, radiologists, and surgeons…If there were links between departments… the process could be shortened…” [P#24, Male, 30years, Physician]
Theme Three: Policy and strategy related	Limited government support	“…Up to now, we have not seen any support from anyone…The government needs to give this proper attention… ensure diagnostics are available…There is a significant gap in terms of supply and availability of reagents…The attention given to it is very minimal…” [P#12, Male, 35years, Nurse]
	Lack of contextual screening models	“…We can’t do endoscopy screening for everyone; we need simpler, locally adapted tools…” [P#20, Male, 38years, Oncologist]
Theme Four: Emotional and ethical challenges in care		“…We treat the mental state of the patient… tell them it’s not the end …Simply silently providing care when a patient cannot afford it is not correct… When patients recover well, that is good…It connects you closely with the Creator… This is very painful…” [P#24, Male, 30years, Physician]
Theme Five: research and evidence gap		“…For instance, research is lacking to show that screening has a significantly high survival benefit. That’s why those who create policies need to work thoroughly on this issue…” [P#14, Male, 37years, Oncologist]

### Theme I: Patient-related factors

#### Sub-theme one: late presentation and delayed health-seeking

Most patients sought medical care only after their disease had progressed to advanced stages (Stage III or IV), when symptoms such as severe dysphagia, weight loss, and food obstruction became severe. The vague and non-specific nature of early symptoms often led patients to interpret them as minor gastrointestinal illnesses rather than warning signs of cancer. Consequently, many initially sought care from nearby health centers, private clinics, or traditional healers.

*“… Often*,* this illness does not cause pain… they say*,* ‘We were expecting to go today*,* tomorrow*,* and the day after tomorrow’ so the stage of the cancer was advanced*,* stage three or four…Many say they went to nearby health centers*,* private clinics and used traditional methods… they say*,* ‘we thought this will heal…” (*P#13, Male, 23year, Nurse).

Several participants delayed seeking medical care because of competing work demands, limited awareness of esophageal cancer, and underestimating the seriousness of their symptoms. Collectively, these factors reduced the perceived urgency of seeking care, resulting in delayed presentation until symptoms became severe. In retrospect, many participants viewed these delays as missed opportunities that they believed may have affected their treatment outcomes.

*“…The reason I failed to realize it quickly was just since I am merchant*,* I was busy…Within those three months*,* I didn’t know that it was this disease…”* (P#2, 53years, Female, EC Stage IV patient).

#### Sub-theme two: traditional and religious care seeking

Participants’ narratives indicate that the use of traditional medicine was deeply embedded in cultural beliefs and perceptions of illness. Temporary improvement following traditional treatments often strengthened confidence in these remedies, leading patients to continue seeking traditional care rather than pursuing medical care. This interaction between cultural beliefs, perceived treatment effectiveness, and delayed healthcare seeking contributed to late presentation for diagnosis.

*“… they turn to traditional medicine…People often believe that traditional medicine is ‘good medicine given to us’…Some believe it is caused by a curse…”* [P#23, Female, 45years, Nurse].

*“….The traditional medicine was what I preferred…Something like a small bean*,* that’s the method*,* it’s called ‘hoofee’… Yes*,* they (healers) said it would heal me… It actually healed me. It healed me at that time*,* and now it has returned again…In my view*,* traditional medicine is good; I only went there…”* [P#4, 84years, Male, EC Stage IV patient].

Participants also described harmful traditional practices that were used in attempts to treat esophageal symptoms. These included thermal cauterization (*gubamuu/waaddamuu*), the insertion of heated metal through a tube-like plant locally known as *Shanbaqqoo* into the esophagus, and chest burning (*koobaa*). According to participants, these practices sometimes resulted in severe complications, including tracheoesophageal fistula, and delayed access to appropriate medical care by diverting patients from biomedical treatment.

*“…Some even said they tried traditional healing…They say they put some tube plant (Shanbaqqoo) through the mouth down to the esophagus and then insert hot metal inside to burn it…We’ve seen two or three such cases… It even caused a fistula…it connected the esophagus and the trachea (Tracheoesophageal fistula)… They also apply burning on their chest*,* called ‘Koobaa’…”* [P#19, Male, 38years, Physician].

The findings indicate that religious beliefs shaped participants’ perceptions of illness and appropriate treatment. For some patients, strong faith in divine healing led them to prioritize prayer, holy water, and other religious practices over biomedical care, particularly during the early stages of illness. In some cases, this resulted in delayed acceptance of the diagnosis or postponement of medical treatment, suggesting that religious beliefs interacted with healthcare-seeking decisions and contributed to delayed diagnosis. However, other participants described using religious practices alongside biomedical care, indicating that faith did not universally discourage engagement with health services.

“*…Some Muslim patients may rely on prayer… believing it may heal or prevent death…Orthodox Christian patients perform prayers*,* holy water rituals… thinking these practices will make them healthy…These cultural and religious approaches sometimes delay medical care…”* [P#5, Male, 30years, Nurse].

*“…Because I was deeply religious and believed strongly in the ‘holy Tabot’ (church shrine)*,* I refused and said*,* ‘I’ll be healed with holy water.’… I belief it (holy water) will cure me. I was thinking it expels the disease… because I strongly believe in it; it will work for me… Even two years ago*,* I had some illness… but I didn’t go to a health facility… When I was first examined two years ago*,* I refused the diagnosis and went for holy water…”* [P#10, Female, 60years, EC Stage IV Patient”.

#### Sub-theme three: cancer-related fear, stigma, and fatalistic beliefs

Participants described fear, stigma, and fatalistic beliefs as interconnected factors that influenced responses to cancer diagnosis and treatment. The perception that cancer inevitably leads to death created fear of diagnosis and reduced patients’ willingness to seek timely medical care or continue treatment. This fear was reinforced by stigma, as some participants associated cancer with a curse, prompting patients to conceal their diagnosis or request that healthcare providers withhold it from family or community members. Fatalistic beliefs that cancer was incurable further discouraged treatment adherence, with some patients discontinuing medication or avoiding follow-up care because they believed that medical intervention could not alter the outcome. Consequently, many participants reported that patients experienced shock upon receiving a cancer diagnosis, which further delayed acceptance of the disease and engagement with appropriate care.

*“…People fear cancer when it is mentioned… Some believe cancer is caused by a curse…”* [P#7, Female, 29years, Physician].

“…*When cancer is mentioned*,* people become frightened…There is a strong belief that anyone who has cancer will inevitably die…Some patients run away immediately when they hear the word…Almost all of them hide it… They ask us to keep it secret…”* [P#5, Male, 30years, Nurse].

*“…The day they finally gave me a clear diagnosis*,* I was shocked and fainted… From the day I knew about this illness*,* I haven’t been able to get out of bed… If I die with this hair loss… what good is it for me? …”* [P#1, Female, 43years, EC Stage III patients].

Participants’ perspectives indicated that fear of surgery, chemotherapy, and endoscopic procedures discouraged engagement with biomedical care. As a result, some patients refused or discontinued recommended treatments, postponed medical care, or turned to traditional or spiritual remedies, contributing to delayed treatment and presentation at more advanced stages of disease.

“*…Many people are afraid when they hear the word ‘surgery’… They refused surgery…”* [P#23, Male, 45years, Nurse].

*“… After undergoing it once*,* patients often don’t want to repeat it …The patient refuses and says*,* ‘No*,* don’t repeat it; that tube doesn’t go into my mout*h (*Endoscopic procedures*)’*…”* [P#19, Male, 31years, Nurse].

*“…We counseled her several times*,* but she didn’t want chemotherapy… the disease spread and she came back unable to walk*…”[P#20, Male, 38years, Oncologist].

*“…It wasn’t because I lacked money*,* but because I was afraid of the surgery …”* [P#3, Female, 50years, EC Stage III patient].

The findings suggest that fatalistic beliefs discouraged timely healthcare seeking by reducing patients’ confidence in the benefits of early diagnosis and treatment. The perception that cancer was incurable, reinforced by observing poor outcomes among other patients in their communities, led some participants to delay seeking medical care until their disease had progressed to an advanced stage.

*“…Many patients just accept their fate instead of coming early*,* thinking it’s hopeless…”* [P#14, Male, 37years, Oncologist].

*“…We were spending money for nothing…They said they’d call*,* and we also tried calling [for test results]…”* [P#15, Male, 82years, EC Stage III patient].

*“…In the past*,* people who got it would just die; we would see it with our own eyes…Even one of my relatives had it…”* [P#10, Female, 60years, EC Stage IV patient].

#### Sub-theme four: lack of awareness and education

The findings suggest that limited awareness among both patients and healthcare providers was a major barrier to early diagnosis. Patients often lacked knowledge of esophageal cancer and misinterpreted early symptoms as less serious conditions, delaying healthcare seeking. Among healthcare providers, limited opportunities for continuing professional training and low clinical suspicion, partly because esophageal cancer was perceived as an uncommon disease, hindered its early recognition and timely referral. Inadequate communication about the diagnosis further reduced patients’ understanding of their condition. Collectively, these factors delayed the recognition, referral, and diagnosis of esophageal cancer.

*“…I was the first to receive that training in this hospital…I have not received any training since then…We are still working with the knowledge we had six years ago…I personally feel urgent training is much needed…”* [P#5, Male, 30years, Nurse].

*“…Cancer is considered as something distant. People think of cancer as if it doesn’t exist…Healthcare workers do not describe cancer separately or explicitly…Rather than only focusing on expanding diagnostic centers… it’s more important to raise awareness among healthcare workers…”* [P#20, Male, 38years, Oncologist].

*“…I felt pain in my chest*,* and I had many difficulties*,* and I thought maybe it was from all the stress. I mistook it*,* I just thought it was heart-related…I had no idea it was something else… I just didn’t know…”* [P#10, Female, 60years, EC Stage IV patient].

*“….Do I know anything? They are the ones who know*… *They told me this is the kind of disease that brings people to the grave…I didn’t know this disease before. Even now*,* I don’t understand it well… ”* [P#8, Male, 65years, EC Stage IV patient].

#### Sub-theme five: financial constraint

Participants’ narratives indicate that financial constraints hindered timely diagnosis and treatment by limiting patients’ ability to afford diagnostic investigations, treatment, and follow-up care. To cope with these costs, some participants postponed medical care while borrowing money, selling personal belongings, or waiting for seasonal income after harvest. The high cost of diagnostic investigations, particularly CT scans and endoscopy, together with additional expenses for transportation, food, accommodation, and caregivers, further prolonged the pathway to diagnosis. Consequently, some patients postponed recommended investigations, interrupted the diagnostic process, or discontinued treatment until they secured financial support through loans, donations, or other sources. Overall, these economic challenges reduced access to timely diagnosis and continuity of care, contributing to late-stage presentation and treatment interruption.

*“…patients are forced to go back home and sell their belongings…It can take as much as 30*,* 40*,* or 50 thousand just to get diagnosed…patients are spending 100*,* 200*,* or even 300 thousand birr in total…Some are unable to afford the costs…” [P#12*,* Male*,* 35years*,* Nurse]*.

*“…When you tell them that a CT scan is necessary*,* they go back home to search for money and come back later… Many patients… during the rainy season can’t afford the expenses… They only come when the harvest season arrives… They come after borrowing money*,* begging*,* or collecting donations through the mosque…” [P#20*,* Male*,* 38years*,* Oncologist]*.

“…*Transportation cost… one person cannot go alone…There are fees for tests… food… many expenses are incurred… ” [P#23*,* Female*,* 45years*,* Nurse]*.

*“…I don’t even have money for medical tests… we sold the land*,* did other things… people who have seen this situation pitied me and asked for help*….” [P#1, Female, 43years, EC Stage III patient].

*“…While trying to sort all this out*,* I lost all my wealth…”* [P#8, Male, 65years, EC Patient].

#### Sub-theme six: geographic access issues

Participants described that geographic barriers contributed to delays in diagnosis. Limited availability of specialized diagnostic services at lower-level health facilities forced many rural patients to travel to distant referral hospitals, often after being referred between multiple providers. This prolonged the pathway to diagnosis while increasing transportation, accommodation, and other indirect costs, making it more difficult for patients to access and remain engaged in care.

*“…CT scan wasn’t available… so we used to refer patients to a town called Dodola*. *There’s also transport cost involved. Even there*,* accurate staging is not done properly…”* [P#20, Male, 38years, Oncologist].

*“…one person cannot go alone; if you look at it personally*,* a patient often travels with two or three people for diagnosis…”* [P#23, Female, 43years, Nurse].

*“… After coming here (to Adama)*,* I went to see a Doctor at private clinic. They told me the doctor wasn’t here and sent me to Minilik Hospital …”* [P#9, Female, 40years, EC Stage III patient].

#### Sub-theme seven: family influence and support

Participants identified family support, particularly from patients’ children, as an important facilitator of early diagnosis. By encouraging timely healthcare seeking and providing practical and emotional support throughout the diagnostic process, family members helped patients overcome delays in accessing diagnostic services.

*“…It was actually one of my children*,* who told me to go…They said*,* you can’t even grind grain anymore… So I followed their advice and came to stay with my children … you’re making your children cry… The children don’t spare any effort… They said it won’t be left behind…Only one of my children refused. …”* [P#10, Female, 60years, EC Stage IV patient].

### Theme II: health system related

#### Sub-theme one: misdiagnosis of the disease

Participants described symptom misinterpretation and misdiagnosis as important barriers to early diagnosis. Early symptoms of esophageal cancer, such as dysphagia and dyspepsia, were frequently attributed to benign conditions, including gastritis and peptic ulcer disease, by both patients and primary healthcare providers. Consequently, many patients sought care repeatedly at public and private health facilities without receiving appropriate investigations or referral for suspected esophageal cancer. This low clinical suspicion, particularly at lower-level health facilities, prolonged the pathway to diagnosis and contributed to presentation at advanced stages of the disease.

*“…Symptoms may resemble indigestion (dyspepsia)… Because of this*,* in the early stages*,* the disease often remains undetected…In lower-level hospitals*,* patients are often treated for peptic ulcer disease (PUD)… Around 95% of the cases I collected data on were in stages III and IV…”* [P#20, Male, 38years, Oncologist].

*“…I kept being told it was just gastritis… Whenever they listen to me with a stethoscope*,* they say it’s gastritis. Wherever they press on my body*,* they say it’s gastritis …”* [P#1, Female, 43years, EC Stage III patient].

*“…One doctor in Robe told me*,* ‘You are fine*,* there is nothing wrong with you*,* go home’…One of them told me it was something related to lung disease…He said it came from gastritis and gave me drug for it…Injection*,* syrup*,* and tablet…”* [P#15, Male, 82years, EC III patient].

#### Sub-theme two: referral system dalliance and complexity

Participants described inefficiencies within the referral and diagnostic system as a major barrier to early diagnosis. Delayed or inappropriate referrals from lower-level health facilities, together with fragmented diagnostic services, prolonged the pathway to diagnosis. Many patients moved between private clinics, health centers, and referral hospitals before receiving a confirmed diagnosis, often undergoing repeated referrals for endoscopy, CT scans, and laboratory investigations. Limited coordination between health facilities and interruptions in diagnostic services further delayed access to essential diagnosis, requiring patients to travel repeatedly to distant centers. These system-level challenges prolonged waiting times, delayed diagnostic confirmation, and contributed to late presentation.

*“…The process (diagnosis) is long… referred for an endoscopy… then sent elsewhere… then referred again for a CT scan… Even after taking a sample… it may take up to a month… If it operates one week*,* the next week it stops*,* or they are referred elsewhere… Patients have to travel to other places just to get this disease diagnosed…”* [P#19, Male, 31years, Nurse].

*“… When you refer*,* they sometimes send the patient back to you …”* [P#20, Male, 38years, Oncologist].

*“…The hospital in Gobessa referred me to Asella. Then Asella referred me here*,* to Adama Hospital… then to Tikur Anbessa Hospital…To find out what the disease was*… *After going through all this… I finally found something…*” [P#8, Male, 65years, EC Stage IV Patient].

*“…Then they told me to go to Dodola…We had even gone to Sodo and Gurage…After Dodola*,* we also went to Borana and Wondo Hospital…The test result came back after a month and fifteen days. What killed me is that the result didn’t come on time… ”* [P#15, Male, 82years, EC Stage III patient].

#### Sub-theme three: limited diagnostic services

Limited availability and poor functionality of diagnostic services emerged as one of the major health system barriers to the early diagnosis of esophageal cancer. Diagnostic investigations were often unavailable, inconsistent, or interrupted, particularly in rural and primary healthcare settings. Many facilities lacked essential diagnostic equipment, including endoscopy, CT scans, and immunohistochemistry (IHC), while existing equipment was sometimes non-functional, even in tertiary hospitals. Consequently, patients were frequently referred to distant specialized centers, such as Adama, Asella, Goba, Addis Ababa and elsewhere, to obtain diagnostic investigations. These repeated referrals, together with prolonged laboratory turnaround times for pathology and other investigations, extended the pathway to diagnosis. Participants perceived these challenges as reflecting broader weaknesses in the healthcare system and contributing to geographic inequities in access to diagnostic services, ultimately resulting in delayed diagnosis and more advanced disease at presentation.

*“…The availability of diagnostic centers is currently limited in the country… Diagnoses like CT scans are not found…It exists*,* but sometimes it doesn’t work…The diagnosis called Immunohistochemistry (IHC) … is often sent outside for identification…”* [P#13, Male, 23years, Nurse].

“… *There are no proper investigations or specialized doctors in our area…The local doctor treat people casually*,* so I didn’t expect proper care there…For this illness*,* they said*,* ‘We can’t treat this; you must go to the hospital…”* [P#22, Male, 83years, EC Stage IV Patient].

*“…The tools available here couldn’t see inside the esophagus…He advised me to go to Hawasa for proper diagnosis*…” [P#17, Male, 54years, EC Stage III patient].

#### Sub-theme four: shortage of trained personnel

Participants identified shortages of specialized healthcare professionals and inadequate training as the other major barriers to the early diagnosis and management of esophageal cancer. The limited availability of oncologists, gastroenterologists, and healthcare providers trained in endoscopy, particularly outside major urban centers, restricted access to specialized diagnostic services. In addition, participants reported that pre-service education and continuing professional development did not adequately equip many frontline healthcare providers to recognize the early signs of esophageal cancer. Training opportunities also varied across professional groups, with nurses, pharmacists, and laboratory personnel receiving less cancer-related training than physicians. Consequently, limited diagnostic competence and low clinical suspicion among frontline providers contributed to delayed recognition, inappropriate management, and late referral of suspected cases.

*“…We only have one oncologist visiting from Tikur Anbessa once in a while…Most of us didn’t learn much about esophageal cancer in medical school; it’s overshadowed by other cancers…” [*P#14, Male, 37years, physician*]*.

*“… In other departments like nursing*,* pharmacy*,* or laboratory*,* at the undergraduate level*,* they don’t study [cancer] thoroughly…They often don’t immediately suspect cancer; instead*,* they suspect other illnesses first…The awareness I had before studying this master’s program and what I have now is very different…”* [P#18, Male, 30years, Nurse].

*“…Honestly*,* the hospital here is in poor condition … There is no doctor who can properly identify the illness…”* [P#21, Male 54years, EC Stage IV patient].

*“…The doctor here treats people casually…I only went for a different issue*,* not for this one…”* [P#22, Male, 83years, EC Stage IV patient].

#### Sub-theme five: health insurance coverage

Participants described health insurance as an important facilitator of timely diagnosis by reducing out-of-pocket diagnostic costs. However, limited insurance coverage for essential diagnostic investigations, including CT scans and endoscopy in settings where these services were unavailable locally, meant that many patients still faced substantial financial barriers. As a result, some delayed diagnostic investigations until they could afford the associated costs.

“…*Those who have insurance do not worry much…Even though insurance is used… still do not get access at most tertiary hospital level…If their health insurance were properly organized*,* it would help a lot…Lack of insurance means not having money…” [P#24*,* Male*,* 30Years*,* Physician]*.

*“…Some patients benefit from insurance*,* which is a good thing…Insurance does not cover CT scans…It only covers X-rays*,* ultrasound*,* blood tests*,* and biopsies…Endoscopy is not available locally…”* [P#6, Male, 34years, Nurse].

*“…If the diagnosis system is available*,* insurance will cover it… Most of the time*,* they are not available…”* [P#20, Male, 38years, Oncologist].

#### Sub-theme six: need for multidisciplinary and integrated cancer care

Participants highlighted multidisciplinary collaboration as a key facilitator of timely diagnosis and management of esophageal cancer. Coordinated teamwork among surgeons, pathologists, radiologists, oncologists, and other specialists was perceived to improve communication, accelerate diagnostic decision-making, and facilitate timely referral and treatment. Participants believed that stronger interdepartmental coordination could streamline the pathway to diagnosis and improve patient outcomes.

*“….We*,* with the surgeons*,* work closely together… we cannot work alone…Links between departments should be strengthened… we should function as a single unit…”* [P#12, Male, 37years, Nurse].

*“…We cannot work alone…We need pathologists*,* radiologists*,* and surgeons…If there were links between departments… the process could be shortened…”* [P#24, Male, 30years, Physician].

### Theme three: policy and strategy related

#### Sub-theme one: limited government support

Participants perceived limited government commitment as a major system-level barrier to the early diagnosis and management of esophageal cancer. Weak policy support, inconsistent funding, and the absence of disease-specific strategies limited the prioritization of esophageal cancer within the national health system. Participants also highlighted the lack of a national cancer registry, which constrained disease surveillance, service planning, and resource allocation. In addition, frequent medication stock-outs, shortages of laboratory reagents, inadequate infrastructure, and inconsistent support from development partners disrupted the availability of diagnostic and treatment services. Collectively, these health system and policy gaps reduced the capacity to provide timely, continuous, and equitable cancer care, contributing to delays in diagnosis and treatment.

*“…Up to now*,* we have not seen any support from anyone…The government needs to give this proper attention… ensure diagnostics are available…There is a significant gap in terms of supply and availability of reagents…The attention given to it is very minimal…”* [P#12, Male, 35years, Nurse].

*“…There’s no guideline or cancer registry for esophageal cancer like there is for cervical cancer … a standardized and centralized guideline would be very helpful…To tell the truth*,* based on its prevalence*,* especially in the Goba (Bale) and Arsi areas; nothing is being done…The government has no motivation or involvement…”* [P#20, Male, 38years, Oncologist] *“….As a country*,* it can be said that we don’t do much screening. The most common screening done is only for cervical cancer…If support were available*,* it should ideally begin with diagnostics…Even patients who could be cured often cannot receive treatment due to lack of resources …”* [P#18, Male, 30years, Nurse].

#### Sub-theme two: lack of contextual screening models

Participants considered globally established screening models impractical in Ethiopia because of limited resources and healthcare infrastructure. Instead, they believed developing context-specific screening approaches for primary healthcare settings and implementing targeted screening among high-risk populations, particularly older adults from high-incidence areas, with dysphagia, smokers, alcohol users, or those with a family history, to facilitate earlier detection of esophageal cancer.

*“…We can’t do endoscopy screening for everyone; we need simpler*,* locally adapted tools…”* [P#20, Male, 38years, Oncologist].

*“…If screenings were available*,* it would be very helpful…Screening is especially important for people aged forty-five and above…”* [P#19, Male, 31years, Nurse].

#### Theme four: emotional and ethical challenges in care

Participants reported that caring for patients with advanced esophageal cancer placed a considerable emotional burden on healthcare providers. Poor outcomes associated with late presentation, together with patients’ inability to access or continue care because of poverty and health system failures, contributed to frustration, helplessness, and moral distress. These experiences underscored the broader impact of delayed diagnosis on both patients and healthcare providers.

*“…We treat the mental state of the patient… tell them it’s not the end …Simply silently providing care when a patient cannot afford it is not correct… When patients recover well*,* that is good…It connects you closely with the Creator… This is very painful…”* [P#24, Male, 30years, Physician].

*“…If I die with this hair loss… what good is it for me?…Honestly*,* I no longer went to that private doctor who treated me before and made things worse*…” [P#1, Female, 43years, EC Stage III patient].

*“…How can you say I’m fine when I feel I’m dying?…I cried and left…You wouldn’t understand unless you were sick yourselves*…” [P#15, Male, 82years, EC Stage III].

#### Theme five: research and evidence gap

Participants highlighted the need for locally relevant research to strengthen the early diagnosis of esophageal cancer. They believed that limited evidence on the disease’s causes, risk factors, and the effectiveness of screening has hindered the identification of high-risk populations and constrained the development of context-specific screening strategies and evidence-informed policies. Participants emphasized that generating local evidence would improve understanding of disease patterns, inform targeted screening programs, and support policy decisions aimed at facilitating earlier diagnosis.

*“…Even through research*,* such issues can be identified and studied…I think it would be good to conduct research in their local areas… ”* [P#13, Male, 23years, Nurse].

*“…For instance*,* research is lacking to show that screening has a significantly high survival benefit. That’s why those who create policies need to work thoroughly on this issue…”* [P#14, Male, 37years, Oncologist].

“… *If research were conducted to understand why… that would be good…There are only suspicions; there is no confirmed reason… ”* [P#24, Male, 30years, Physician].

## Discussion

The study revealed that early diagnosis of esophageal cancer was influenced by multiple interconnected barriers and facilitators operating at the patient, health system, and policy levels. In addition, emotional and ethical challenges in cancer care and gaps in research and evidence emerged as important cross-cutting factors influencing early diagnosis.

At the patient level, late presentation and delayed healthcare seeking, reliance on traditional and religious healing practices, cancer-related fear, stigma and fatalistic beliefs, limited awareness and education, financial constraints, and geographic barriers emerged as key obstacles to early esophageal cancer diagnosis. In contrast, family influence and support were identified as important facilitators of early diagnosis. Within the health system, misdiagnosis, fragmented referral pathways, limited diagnostic services, and shortages of trained healthcare professionals were identified as major barriers to the early diagnosis of esophageal cancer. Conversely, health insurance coverage and multidisciplinary, integrated cancer care facilitated early diagnosis. At the policy level, inadequate government support and the lack of locally relevant screening models left early detection initiatives without the necessary structural foundation. Compounding these issues were a lack of locally relevant research and evidence to guide prevention and screening, alongside emotional and ethical challenges that affected both patients and providers. Together, these interconnected barriers formed a complex web that consistently resulted in patients being diagnosed at advanced, often untreatable, stages of esophageal cancer (Fig. 1).

The finding that most patients presented at advanced stages (III or IV) was consistent with studies from other low- and middle-income countries (LMICs)^47^. Research from Zambia, Kenya, Tanzania, and Malawi reported that esophageal cancer patients presented with late-stage^48–52^. Patients normalize their early symptoms as harmless, delaying care-seeking. Although they later blamed themselves, delays were not solely individual failings but are influenced by broader factors such as symptom interpretation, social context, and healthcare access barriers^53^. In support of these, low awareness of esophageal cancer symptoms among patients and providers was a fundamental barrier to early diagnosis, consistent with studies in Ethiopia^54^ and China^55^. Patients often misinterpreted symptoms as minor ailments^54^, while providers’ lack of updated training contributed to diagnostic delays^56^. Educational status also influenced timely diagnosis, as reported in a systematic review of low- and middle-income countries (LMICs)^57^.

Likewise, financial barriers, geographic inequities, and limited diagnostic infrastructure considerably impeded early diagnosis of esophageal cancer. Patients delayed care due to the high cost of diagnostic investigations, some selling belongings or waiting for harvest to afford diagnosis and treatment^57^. Beyond medical expenses, transportation, food, and accommodation costs added to the burden, particularly when patients required companions^58^. Similar findings have been reported in Ethiopia^54,58^, Uganda^58,59^, and Kenya^49,60^. Geographic disparities further exacerbated diagnostic delays by increasing travel distances and the cost of accessing care, as specialized diagnostic services were concentrated in urban centers such as Goba, Adama, Asella, and Addis Ababa. This finding is consistent with the African Esophageal Cancer Consortium, which highlighted that limited opportunities for early detection and diagnostic services across the East African esophageal cancer corridor contribute to delayed diagnosis and poor patient outcomes^14^. Rural patients described lengthy diagnostic journeys, often traveling between facilities over several months before receiving a confirmed diagnosis^54^. Furthermore, essential diagnostic tools (endoscopy, CT scan, biopsy and immunohistochemistry) were frequently unavailable, non-functional, or unaffordable in the area, reflecting the limited diagnostic capacity in LMICs^61^.

The study also found that cancer-related fear, stigma, and fatalistic beliefs emerged as interconnected barriers to early diagnosis by reducing patients’ confidence in the benefits of timely diagnosis and treatment. Participants frequently perceived cancer as an inevitable death sentence, making diagnosis appear futile and discouraging healthcare seeking. This pattern reflects the concept of cancer fatalism described by Keeley et al.^62^ and is consistent with evidence showing that fatalistic beliefs were associated with lower participation in cancer diagnosis behaviors^63^. Similar perceptions have been reported among patients with esophageal cancer in China, where a diagnosis often evoked an immediate fear of death^64^, supporting the observation that cancer was widely equated with death^65^. Fear also extended to diagnostic procedures and treatment, with some patients refusing surgery, chemotherapy, or repeat endoscopy because of anticipated discomfort or perceived danger, thereby interrupting the diagnostic pathway^66^. Furthermore, patients’ requests for diagnosis secrecy reflect deep cancer stigma, consistent with evidence linking stigma to diagnosis concealment and care delays^67^. This stigma contributed to feelings of rejection, self-hatred, worthlessness, and hopelessness, with some patients considering treatment withdrawal^68^.

Another important finding was that that delayed diagnosis within the health system resulted from the interaction of weaknesses across the diagnostic pathway rather than a single system failure. Misdiagnosis of early esophageal cancer symptoms as gastritis, peptic ulcer disease, or dyspepsia represented the first barrier, reflecting low clinical suspicion among frontline healthcare providers and the overlap between early cancer symptoms and common gastrointestinal conditions. Similar challenges have been reported in other low- and middle-income countries, where limited diagnostic capacity and low awareness among primary care providers contribute to missed or delayed cancer diagnoses^57^. In Ethiopia, the high prevalence of Helicobacter pylori*-*related dyspepsia may further reinforce this diagnostic challenge by increasing the likelihood that early esophageal cancer symptoms are attributed to benign conditions^74^.

Consequently, patients often entered prolonged and fragmented referral pathways, moving between health centers, private clinics, district hospitals, and referral centers before receiving a definitive diagnosis. This pattern reflects broader weaknesses in the Ethiopian referral system, where poor coordination, multiple referral points, and service interruptions prolong access to specialist care^75–77^. This finding is supported by previous studies showing that multiple factors compromise referral system performance^78^, and that each additional referral represents a potential point of delay or patient dropout, cumulatively contributing to disease progression^79^.

These challenges were further compounded by shortages of oncologists, gastroenterologists, pathologists, and other trained healthcare professionals, together with limited cancer-specific training among frontline providers. Insufficient knowledge and low clinical suspicion reduced opportunities for early recognition and appropriate referral, while inadequate training among nurses, pharmacy personnel, and laboratory professionals weakened multidisciplinary cancer detection. These findings are consistent with previous studies demonstrating that strengthening the knowledge and skills of frontline healthcare workers improves early cancer recognition, timely referral, and opportunities for early diagnosis and prevention^14,69^.

Moreover reliance on traditional and religious healing before seeking biomedical care reflected deeply rooted cultural interpretations of illness and interacted with broader health system barriers to delay diagnosis. Participants described seeking holy water, prayer, and traditional healers because they believed cancer was caused by a curse or could be cured through spiritual intervention, beliefs that contrast with the biomedical understanding of the disease. Similar patterns of medical pluralism have been reported across Africa^70–72^ and Pakistan^73^. Importantly, participants’ narratives suggested that reliance on these practices was not solely driven by cultural beliefs but was also reinforced by limited access to affordable and effective biomedical services. Harmful traditional procedures, including thermal cauterization (“gubamuu/waaddamuu”) using heated metal inserted through a tube plant (“Shanbaqqoo”) and chest burning (“Koobaa”), were reported to cause serious complications such as tracheoesophageal fistula, further delaying access to appropriate medical care. Similar practices have been documented elsewhere in Ethiopia^54,74^.

On the other hand, family support and influence, health insurance coverage, and multidisciplinary, integrated cancer care emerged as important facilitators of early esophageal cancer diagnosis. Participants’ described that support from family members, particularly adult children, encouraged timely healthcare-seeking and helped overcome diagnostic delays, consistent with social support theories that emphasize the role of interpersonal relationships in shaping health seeking behaviors^75^. Although health insurance reduced some financial barriers, its impact was constrained by incomplete benefit packages, limited coverage of essential diagnostic services, and restricted access to specialized cancer care. The finding that uninsured patients “may not return at all” underscores the detrimental effect of out-of-pocket costs on continuity of care, particularly in the context of low community-based health insurance coverage in Ethiopia^76^. Previous studies have similarly shown that health insurance improves access to care when benefit packages adequately cover essential services and meet population needs^77–79^. Participants also emphasized the importance of stronger interdepartmental collaboration, and multidisciplinary cancer care, reflecting global evidence that integrated care models improve diagnostic efficiency, treatment planning, and patient outcomes^80^.

Participants perceived esophageal cancer as a neglected disease, reflecting gaps in national cancer control, including the absence of a cancer registry, disease-specific guidelines, organized screening programs, and sustained policy support. These findings underscore the need for stronger government commitment and align with recommendations for integrating cancer control into national health policies in Sub-Saharan Africa^81^. Participants also emphasized the need for context-specific screening strategies and locally generated evidence to identify high-risk populations and inform effective early detection policies in resource-constrained settings^82,83^.

Beyond these structural challenges, participants described the considerable emotional and ethical burden associated with esophageal cancer. Healthcare providers expressed frustration and moral distress when patients were unable to access timely diagnosis and treatment because of health system constraints, while patients reported shock, fear, hopelessness, and despair following diagnosis.

### Strengths and limitations

This research has several inherent limitations. Participants’ responses were shaped by their personal experiences, beliefs, and emotions, which may introduce bias and limit transferability. Recall bias may affect the credibility of timelines and specific case details, while social desirability bias may have influenced responses on sensitive topics such as religion, traditional medicine use, stigma and fear. Additionally, the study did not include interviews with traditional healers, religious leaders, or policy personnel, which limited the opportunity to capture their perspectives and experiences. Member checking was not conducted with esophageal cancer patients. Although efforts were made to establish rapport and encourage open discussion, participants’ responses may have been influenced by the interviewer’s professional background or perceived role. Despite these the study showed rich, in-depth qualitative data capturing multiple perspectives, from both late-stage patients and frontline healthcare providers, on barriers and facilitators to early esophageal cancer diagnosis. The inclusion of participants from diverse geographic areas and health system levels enhances the comprehensiveness of findings, while thematic analysis enabled the identification of interconnected factors across multiple levels of influence.

## Conclusions

In conclusion, this study demonstrated that early diagnosis of esophageal cancer in Central Oromia (Arsi, Bale, East Bale, East Shewa, and West Arsi) is influenced by multiple interconnected barriers and facilitators operating at the patient, health system, and policy levels. Emotional and ethical challenges in cancer care, together with gaps in research and evidence, also emerged as important cross-cutting factors. Addressing these complex barriers requires a coordinated, multi-level approach that integrates community awareness, health system strengthening, continuing professional training for healthcare providers, multidisciplinary cancer care, context-specific research, and policy reforms that prioritize esophageal cancer, strengthen referral systems, and expand access to affordable, decentralized diagnostic services. Such interventions are essential to promote earlier diagnosis and improve survival outcomes in Central Oromia and similar resource-constrained settings. Without sustained action, esophageal cancer will continue to be diagnosed at advanced stages, resulting in poor treatment outcomes and perpetuating the cycle of delayed diagnosis documented in this study.

## Data Availability

The data for this study consist of audio recordings from esophageal cancer patients and healthcare providers working in oncology units, which were transcribed and translated into English. The transcribed and translated versions are available from the corresponding author upon reasonable request.

## Abbreviations

CT: Computed Tomography
DELTAS: Science for Africa Foundation to the Developing Excellence in Leadership, Training and Science in Africa
EDCTP2: European & Developing Countries Clinical Trials Partnership 2
HCP: Healthcare Providers
IHC: Immunohistochemistry
LMIC: Low and middle income countries
MRI: Magnetic Resonance Imaging
NCDs: Non-communicable diseases
NORA: Network for Oncology Research in Sub-Saharan Africa (NORA)
PUD: Peptic Ulcer Disease
SDG: Sustainable Development Goal
